# Right Heart Pulmonary Circulation Unit Response to Exercise in Patients with Controlled Systemic Arterial Hypertension: Insights from the RIGHT Heart International NETwork (RIGHT-NET)

**DOI:** 10.3390/jcm11020451

**Published:** 2022-01-17

**Authors:** Olga Vriz, Paolo Palatini, Lawrence Rudski, Paolo Frumento, Jarosław D. Kasprzak, Francesco Ferrara, Rosangela Cocchia, Luna Gargani, Karina Wierzbowska-Drabik, Valentina Capone, Brigida Ranieri, Andrea Salzano, Anna Agnese Stanziola, Alberto Maria Marra, Roberto Annunziata, Salvatore Chianese, Salvatore Rega, Teresa Saltalamacchia, Renato Maramaldi, Chiara Sepe, Giuseppe Limongelli, Filippo Cademartiri, Antonello D’Andrea, Michele D’Alto, Raffaele Izzo, Nicola Ferrara, Ciro Mauro, Antonio Cittadini, Grünig Ekkehard, Marco Guazzi, Eduardo Bossone

**Affiliations:** 1 Cardiac Centre, King Faisal Specialist Hospital and Research Center, Riyadh 11564, Saudi Arabia; olgavriz@yahoo.com; 2 School of Medicine, Alfaisal University, Riyadh 11533, Saudi Arabia; 3 Department of Medicine, University of Padova, 35122 Padova, Italy; palatini@unipd.it; 4 Azrieli Heart Center and Center for Pulmonary Vascular Diseases, Jewish General Hospital, McGill University, Montreal, QC H3A 0G4, Canada; lawrence.rudski@mcgill.ca; 5 Department of Political Sciences, University of Pisa, 56126 Pisa, Italy; paolo.frumento@unipi.it; 6 Department of Cardiology, Bieganski Hospital, Medical University, 91-347 Lodz, Poland; kasprzak@ptkardio.pl (J.D.K.); wierzbowska@ptkardio.pl (K.W.-D.); 7 Heart Department, University Hospital of Salerno, 84131 Salerno, Italy; fferrara1975@gmail.com; 8 Division of Cardiology, A Cardarelli Hospital, 80131 Naples, Italy; rosangela.cocchia@aocardarelli.it (R.C.); caponevalentina92@libero.it (V.C.); dr.robertoannunziata@gmail.com (R.A.); sasichian@gmail.com (S.C.); makida@alice.it (C.S.); 9 Institute of Clinical Physiology, National Research Council, 56124 Pisa, Italy; gargani@ifc.cnr.it (L.G.); ciro.mauro1957@gmail.com (C.M.); 10 IRCCS Synlab SDN, 80143 Naples, Italy; ranieribrigida@gmail.com (B.R.); andre.salzano@gmail.com (A.S.); cademartirifilippo@gmail.com (F.C.); 11 Department of Respiratory Diseases, Monaldi Hospital, University “Federico II”, 80131 Naples, Italy; annaagnese.stanziola@unina.it; 12 Department of Translational Medical Sciences, “Federico II” University of Naples, 80138 Naples, Italy; albertomaria.marra@unina.it (A.M.M.); salreg25@gmail.com (S.R.); teresa.saltalamacchia@gmail.com (T.S.); renatomaramaldi@gmail.com (R.M.); nicola.ferrara@unina.it (N.F.); antonio.cittadini@unina.it (A.C.); 13 Division of Cardiology, Monaldi Hospital, Second University of Naples, 81100 Naples, Italy; giuseppe.limongelli@unicampania.it (G.L.); mic.dalto@tin.it (M.D.); 14 Department of Cardiology and Intensive Coronary Unit, “Umberto I” Hospital, 84014 Nocera Inferiore, Italy; antonellodandrea@libero.it; 15 Department of Advanced Biomedical Sciences, “Federico II” University of Naples, 80131 Naples, Italy; raffaele.izzo@unina.it; 16 Centre for Pulmonary Hypertension, Thoraxklinik at Heidelberg University Hospital, Translational Lung Research Center Heidelberg (TLRC), German Center for Lung Research (DZL), 69120 Heidelberg, Germany; Ekkehard.Gruenig@med.uni-heidelberg.de; 17 Heart Failure Unit, Cardiopulmonary Laboratory, University Cardiology Department, IRCCS Policlinico San Donato University Hospital, 20097 Milan, Italy; marco.guazzi@unimi.it

**Keywords:** echocardiography stress test, pulmonary pressure, left ventricular diastolic dysfunction

## Abstract

Background. Systemic arterial hypertension (HTN) is the main risk factor for the development of heart failure with preserved ejection fraction (HFpEF). The aim of the study was was to assess the trends in PASP, E/E’ and TAPSE during exercise Doppler echocardiography (EDE) in hypertensive (HTN) patients vs. healthy subjects stratified by age. Methods. EDE was performed in 155 hypertensive patients and in 145 healthy subjects (mean age 62 ± 12.0 vs. 54 ± 14.9 years respectively, *p* < 0.0001). EDE was undertaken on a semi-recumbent cycle ergometer with load increasing by 25 watts every 2 min. Left ventricular (LV) and right ventricular (RV) dimensions, function and hemodynamics were evaluated. Results. Echo-Doppler parameters of LV and RV function were lower, both at rest and at peak exercise in hypertensives, while pulmonary hemodynamics were higher as compared to healthy subjects. The entire cohort was then divided into tertiles of age: at rest, no significant differences were recorded for each age group between hypertensives and normotensives except for E/E’ that was higher in hypertensives. At peak exercise, hypertensives had higher pulmonary artery systolic pressure (PASP) and E/E’ but lower tricuspid annular plane systolic excursion (TAPSE) as age increased, compared to normotensives. Differences in E/E’ and TAPSE between the 2 groups at peak exercise were explained by the interaction between HTN and age even after adjustment for baseline values (*p* < 0.001 for E/E’, *p* = 0.011 for TAPSE). At peak exercise, the oldest group of hypertensive patients had a mean E/E’ of 13.0, suggesting a significant increase in LV diastolic pressure combined with increased PASP. Conclusion. Age and HTN have a synergic negative effect on E/E’ and TAPSE at peak exercise in hypertensive subjects.

## 1. Introduction

Systemic arterial hypertension (HTN) is the most common cardiovascular (CV) risk factor among patients affected by heart failure with preserved ejection fraction (HFpEF) [1,2]. In this regard, left ventricular hypertrophy (LVH) along with abnormal left ventricular (LV) relaxation and left atrial enlargement represents the characteristic “pathologic triad” usually detected by transthoracic Doppler echocardiography (TTE) in hypertensive patients as a direct consequence of the chronically increased LV workload [3,4]. However, it should be noted, in mild-moderate HTN, LV filling pressure and related pulmonary arterial systolic pressure (PASP) may be normal at rest but may show an abnormal and disproportionate increase during exercise [5,6]. From the clinical point of view, an increase in LV filling pressure and PASP during physical activities might explain the dyspnea in those hypertensive patients with well controlled resting blood pressure (BP) and with no signs of HFpEF [5,6].

The aim of the study was to assess the trends in PASP, E/E’ and TAPSE during exercise Doppler echocardiography (EDE) in hypertensive patients vs. healthy subjects stratified by age.

## 2. Methods

### 2.1. Study Population

One hundred and fifty-five consecutive patients with idiopathic uncomplicated mild-to-moderate HTN and one hundred and forty-five healthy subjects were enrolled by 13 RIGHT-NET participating centers [7,8]. Healthy subjects were recruited, as previously reported, among volunteers without structural heart disease on TTE and/or history of any cardiovascular disease and/or any systemic diseases known to affect the cardiovascular system [7,8,9,10,11]. Patients were defined to be hypertensive if office systolic blood pressure (SBP) values were ≥140 mmHg and/or diastolic blood pressure (DBP) values were ≥90 mmHg on three consecutive visits or if they were on anti-hypertensive therapy [4]. Office brachial BP and heart rate (HR) were measured twice in supine position, in the right arm, 10 min apart in a quiet room. All participants underwent full screening for CV disease, including an interview on medical history, use of medications, and lifestyle habits (alcohol intake, smoking, physical activity) [12]. Physical examinations (height, weight, HR and BP) and clinical assessments were conducted according to standardized protocols by trained and certified staff [7,10]. Body surface area (BSA) was calculated according to the DuBois formula (0.20247 × height (m)^0.725^ × weight (kg)^0.425^).

### 2.2. Resting Echocardiographic Doppler Examination

TTE examinations were performed at rest with commercially available equipment on all subjects, according to standardized protocols [7]. All echo-Doppler measurements were obtained according to current American Society of Echocardiography (ASE)/European Association of Cardiovascular Imaging (EACVI) recommendations [13,14,15]. In particular, peak tricuspid regurgitation velocity (TRV) was measured from multiple views (the spectral profile of the TR jet in the RV inflow projection of the parasternal long-axis view, the parasternal short-axis view or the apical 4-chamber view); the highest TRV was used for calculation of RV systolic pressure. The PASP estimation was based on the simplified Bernoulli equation applied to TRV with the addition of estimated right atrial pressure (RAP) based on inferior vena cava (IVC) size and collapse: 5 mmHg was added in all subjects showing IVC < 21 mm diameter and >50% collapsibility to the systolic transtricuspid gradient (PASP = 4 V^2^ + RAP, where V = maximal velocity of tricuspid regurgitation jet) [16]. PASP was assumed to be equal to the RV systolic pressure in the absence of pulmonic stenosis and/or right ventricular outflow tract obstruction. An agitated saline solution was used in cases of poor TRV Doppler signal, such as in the presence of incomplete spectral wave envelope and/or artifacts. Mean pulmonary artery pressure (mPAP) was calculated as 0.6 × PASP + 2 [17]. TAPSE was measured from the 4 chamber views by placing an M-mode cursor through the tricuspid annulus measuring the excursion distance between end-diastole and end-systole (in mm) and with optimal image orientation and alignment to avoid underestimation. The TAPSE/PASP ratio was then estimated [18]. Tissue Doppler velocities (TDI) of the tricuspid annulus were recorded from the apical four-chamber view, by placing the sample volume at the level of lateral corner of the tricuspid annulus, adjusting the spectral pulsed Doppler signal filters within Nyquist limit of 15–20 cm/s, and using the minimal sufficient gain setting to avoid signal blurring.

### 2.3. Exercise Doppler Echocardiography Examination

EDE was performed according to the current recommendations on a semi-recumbent cycle ergometer with an incremental workload of 25 watts every 2 min up to the symptom-limited maximal tolerated workload [7,19,20]. The exercise table was tilted laterally by 20 to 30° to the left. HR (by ECG lead) was continuously monitored, and BP (by sphygmomanometer) was measured at baseline and during the last 15 s of each workload step [7]. Key echocardiographic measurements were acquired at baseline and peak exercise, including left and right ventricular function, valvular gradients, regurgitant flows, left and right heart Doppler derived hemodynamics [PASP, mPAP, pulmonary vascular resistance (PVR), right atrial pressure (RAP) and E/E’ and cardiac output (CO)] [7]. LV diastolic parameters E and A peak velocities (m/sec), their ratio and E wave deceleration time (ms), were measured during low level of exercise (heart rate < 110 bpm). The early (e’) diastolic velocities were measured by TDI at the septal and lateral corner of the mitral annulus and the mean between the two values was calculated. Mitral E velocity, corrected for the influence of relaxation (i.e., the E/mean e’ ratio), was assessed to estimate LV filling pressures [7].

### 2.4. Image Analysis and Quality Control

All participating centers were chosen according to recommended standard operational procedures in terms of data imaging acquisition (operational modes, machine settings), data storage (data format, transfer procedure) and data processing (software used and measurement procedures). Echocardiographic recordings—both at rest and exercise—were reviewed and analyzed offline at each center by a certified operator expert in TTE. A quality control procedure was set to reduce variability among laboratories and operators and to maintain and improve the quality of subsequent collections of data according to the American and European Recommendations and Guidelines [8,9,13,19,20]. In addition, the feasibility of semi-recumbent bicycle EDE for the assessment of right heart-pulmonary circulation function and hemodynamic indices among healthy individuals and patients at risk of developing pulmonary hypertension (PH) was also previously tested and reported [9].

## 3. Statistical Analysis

Data were expressed as mean ± standard deviation and each relative frequency of feasibility into a percentage. Normal distribution of the continuous variables was assessed by the Kolmogorov-Smirnov test. T-test for unpaired observations was used to compare the differences between normal and hypertensive subjects. Differences between normotensive and hypertensive were adjusted by age, sex and BSA. The entire cohort was divided into tertiles of age: group 1, younger than 51 years, group 2, between 51 and 66 years and group 3, older than 66 years. ANOVA was used to compare continuous variables within the groups. Differences were adjusted for confounding factors (sex, BSA, therapy). The chi-squared test was applied to compare categorical variables. A mixed model with estimation method REML was applied to test the effect of age, hypertension state and their interaction adjusted by sex, therapy and corresponding variables at baseline, on the dependent variables at peak exercise. Non-parametric tests were used when assumptions of normality tests were not met. *p* values < 0.05 were considered statistically significant. Data analysis was performed using SPSS (Rel. 11.0, 2002, SPSS Inc., Chicago, IL, USA).

### Ethical Considerations

The study protocol followed the principles of the Declaration of Helsinki and each participating center had obtained both approval from its local research ethics committee and informed consent from each subject. Clinical trials Gov Identifier: NCT03041337.

## 4. Results

Hypertensive patients (78% on antihypertensive therapy) were older (62 ± 12 vs. 54 ± 14.9 years, *p* < 0.0001) and heavier (BMI 27.8 ± 4 vs. 26 ± 3.5 kg/m^2^, *p* < 0.0001) and were less frequently females (27% vs. 40%, *p* < 0.01) as compared to normotensive subjects (Table 1). 

Furthermore, after adjustment for age, sex and BSA, hypertensives have increased LV dimension with LV remodeling, increased PASP, PASP/CI and PVR, higher E/A and E/E’, lower TAPSE/PASP but similar cardiac output (CO) and TAPSE (Table 2).

### 4.1. Exercise Doppler Echocardiography

Hypertensives exercised for a shorter period of time, reached lower peak workload, SBP and HR compared to normotensive healthy subjects even after adjustment for age, sex and BSA. They also reached lower LVEF, SV and CO but higher E/E’. In addition, PASP, PASP corrected by flow (PASP/CI), mPASP and PVR were higher, while TAPSE and TAPSE/PASP were lower in hypertensives (Table 3). 

### 4.2. Analysis by Tertiles of Age

The entire cohort was divided into tertiles of age. In hypertensives, group 1 included 28 patients (mean age 48 ± 8.years), group 2 included 65 patients (mean age 59.6 ± 4.4 years) and group 3 included 62 patients (mean age 73.5 ± 4.4 years). In normotensives, group 1 included 62 subjects (mean age 40.1 ± 9 years), group 2 included 43 subjects (mean age 58 ± 4.5 years) and group 3 included 38 subjects (mean age 72 ± 3.5 years). Age was similar between hypertensive and normotensives within each age group, percentage of males was higher in hypertensives, and BSA was lower in the elderly group of hypertensives (Table 3). Details of general and echocardiographic characteristics at baseline and peak exercise of the hypertensive cohort divided in tertiles of age are reported in Table 4, Table 5 and Table 6. Briefly, baseline echocardiographic data underlined a progressive increase in IVS thickness in diastole according to increase in age and a significant increase in E/E’ as expression of increase of LV end diastolic pressure (LVED). TAPSE was slightly, although significantly, decreasing with age increase. At peak exercise, along with age group increase, the amount of work/exercise time, HR response, LVEF and SV decreased. In addition, TAPSE and TAPSE/PASP decreased, while PASP and also PASP corrected by flow (PASP/CI) increased.

As reported in Table 7, the effect of increase in age combined with the hypertensive state, adjusted by confounders, such as sex, therapy and the corresponding variable at baseline, was responsible for the significant increase in E/E’ and decreased in TAPSE (Supplementary Table S1) meaning that exercise was able to unmask an abnormal increase in LVED pressure and decrease RV longitudinal contraction in hypertensives. No effect of this combination was observed for PASP, TAPSE/PASP or CO.

## 5. Discussion

Echocardiography is an established, user-friendly, low cost, radiation-free imaging technique that can provide abundant information on the structure and function of the heart at rest as well as on exercise. EDE is commonly used in different settings, such as ischemic heart disease [21], valvular heart disease [22] and chronic heart failure [23], including patients with HFpEF who presented with heart failure symptoms during exercise and are characterized as having ‘borderline’ LV diastolic dysfunction [23,24]. Indeed, exercise echocardiography may help rule out HFpEF adding exercise E/e’ data [23,25].

It should be also noted that the additional simultaneous assessment of right heart pulmonary circulation unit function and hemodynamic responses to dynamic exercise may have several clinical diagnostic and prognostic implications [26,27,28,29,30,31,32]. Argiento et al. demonstrated the usefulness of EDE for studying the pulmonary circulation among healthy subjects providing realistic values compared to those obtained by invasive hemodynamic measurements [33,34]. Bossone et al. showed that athletes have higher TRV compared with healthy control subjects both at rest and during exercise related to the higher SV and CO reached [35]. Interestingly, Forton et al., in a cohort of adolescents and adults who underwent EDE, found that pulmonary vascular distensibility coefficient α was inversely related to age at rest as well as during exercise [36]. These results confirm the age-related increase in pulmonary artery pressure (positive correlation) [37,38,39].

### Exercise Doppler Echocardiography in Systemic Arterial Hypertension

As far as we know, there are few studies on EDE in systemic hypertensive patients focusing on the right heart hemodynamics. Vriz et al. [40] studied 25 young, uncomplicated, never treated hypertensive patients at baseline and during bicycle stress test matched for age and sex with a group of healthy subjects The hypertensives had significantly higher mPAP and lower CI values at baseline and at peak exercise compared to healthy subjects, but similar α-index (index of distensibility).

In the present study, when the group of normotensives and hypertensives were divided in tertiles of age, E/E’ ratio, TAPSE, PASP, TAPSE/PASP and CO were similar within the same age group at rest. At peak exercise, on the other hand, there was a significant increase in E/E’ and decrease in TAPSE in hypertensives across age groups, while no significant changes in the normotensive groups were observed. PASP was constantly higher in hypertensives at peak exercise compared to the groups of normotensives.

Echocardiography estimation of E/E’, which is less age-dependent than e’ [41] and is strongly influenced by severity of LVH [42], has been proven to have diagnostic value during exercise [24,25]. Estimation of E/E’ is also reported to have a good correlation with pulmonary capillary wedge pressure (PCWP) at rest and sufficient capacity to predict exercise-induced elevation of PCWP [5,6,43]. In this regard, E/E’ higher than 13 during exercise has been found to have a sensitivity and specificity of 73% and 96% for detecting increased LV diastolic pressure [5]. Moreover, the increase in E/E’ during exercise has a good sensitivity/specificity for prediction of reduced exercise capacity and survival [5,44]. In particular, Shim et al. provided data on the prognostic values of the increase in pulmonary pressure during exercise, but the excessive risk was restricted only to the subjects with combined increase in E/E’ during stress test [45]. In the present study, however, at peak exercise, the oldest group of hypertensive patients had a mean E/E’ of 13, suggesting significant increase in LV diastolic pressure combined with increased PASP.

It should also be highlighted that TAPSE showed, during exercise, a different trend in normal subjects vs. hypertensive ones with a significant progressive decrease as age increased in the later groups. Thus, impaired TAPSE at peak exercise could represent RV-PA uncoupling in hypertensives. In addition, a progressive decrease in TAPSE/PASP as age increased was observed [46].

## 6. Study Limitations

A few limitations of the current study need to be highlighted. Firstly, not all the variables of RV function were considered, such as fractional area change and strain of the RV. As pertains to RV strain, when the multicenter study was started, few centers had echo-machines implemented with strain software and even fewer had a vendor-independent software for 2D strain reading. Nevertheless, the parameters included have been well validated in the context of RV function in the setting of increased afterload, with exercise and in the evaluation of RV-PA coupling. Second, we had a relatively smaller number of healthy elderly subjects that underwent stress testing; however, this number is still substantial when compared with the existing literature. Third, E/e’ ratio may be not accurate for the estimation of LV filling pressures in normal subjects, patients with heavy annular calcification, mitral valve and pericardial disease and coronary artery disease (sampled segments dysfunction). Furthermore, normal cutoff values may vary depending on the measurement site used. In this regard, it should be underlined there is the so called “E/e’ ratio gray zone’’ in which LV filling pressures are indeterminate [15]. Finally, stress invasive hemodynamic tests (gold standard) were not included in the RIGHT-NET [7].

## 7. Conclusions

Hypertensive patients are a heterogeneous group that might require additional investigations to understand the impact of HTN on their disease state. In the present study, at rest, no significant differences in hemodynamics were seen between hypertensive patients and healthy subjects among age groups and, as a consequence, LV filling pressure values were far away from the condition of HFpEF. However, EDE unmasked an evident different trend in LV filling pressure and TAPSE among hypertensive ones. Furthermore, in the elderly hypertensive cohort, the significant increase in LV filling pressure could be partially responsible for the increase in PASP, while the decrease in TAPSE may be a significant marker of RV-PV uncoupling. In summary, age and hypertension have a negative synergic effect on surrogate echocardiographic parameters of LV filling pressure and RV contractility. Further prospective longitudinal studies are needed in order to clearly define a “pre-HFpEF phenotype” along with potential therapeutic interventions.

## Figures and Tables

**Table 1:** General characteristics in normal and hypertensive subjects.

	Normotensive *n* = 145	Hypertensive *n* = 155	*t*-Test	ANOVA Adjusted by Age, Sex, BSA
Age (years)	54 ± 14.9	62 ± 12.0	0.0001	-
M/F	65/58	113/42	0.01	-
BMI (Kg/m^2^)	26 ± 3.5	27.8 ± 4	0.0001	-
BSA (m^2^)	1.97 ± 0.1	1.89 ± 0.2	0.0001	
SBP (mmHg)	123 ± 16	137 ± 20	0.0001	0.0001
DBP (mmHg)	77 ± 10	82 ± 11	0.0001	0.0001
HR (bpm)	75 ± 15	73 ± 13	0.1	0.7
Hemoglobin (dL/L)	14.6 ± 1.8	14.4 ± 1.7	0.3	0.0001
Creatinine (mg/dL)	0.97 ± 0.18	1.0 ± 0.22	0.2	0.9
Glucose (mg/dL)	110 ± 48	117 ± 40	0.4	0.039
Tot Chol (mg/dL)	198 ± 38	177 ± 52	0.07	0.002
HDL (mg/dL)	52 ± 13	49 ± 12	0.3	0.001
Triglycerides (mg/dL)	117 ± 70	119 ± 62	0.9	0.006

Abbreviations: BMI, body mass index; BSA, body surface area; DBP, diastolic blood pressure; HR, heart rate; M/F, Male/Female; SBP, systolic blood pressure.

**Table 2:** Baseline echocardiographic variables in normotensive and hypertensive subjects.

	Normotensive *n* = 145	Hypertensive *n* = 155	*t*-Test	ANOVA Adjusted by Age, Sex, BSA
LVIDD (mm)	47.0 ± 3.9	48.0 ± 7.0	0.1	0.0001
IVSD (mm)	9.7 ±1.2	10.0 ± 2.0	0.0001	0.0001
PWTD (mm)	9.2 ± 1.3	9.2 ± 1.7	0.4	0.0001
LVM (gr)	153.0 ± 36	174.8 ± 69	0.001	0.0001
LVMI (gr/m^2^)	81.9 ± 17	91.8 ± 31	0.001	0.015
RWT	0.35 ± 0.08	0.39 ± 0.09	0.0001	0.0001
LVEF (%)	64.0 ± 7.0	63.0 ± 9.0	0.1	0.023
LAVi (vol/BSA)	22.8 ± 8	26.1 ± 11.7	0.01	0.0001
E/A	1.20 ± 0.5	1.17 ± 0.5	0.2	0.0001
E/E’	7.3 ± 2.5	8.8 ± 3.2	0.0001	0.0001
SV (mL)	69.7 ± 16	71.0 ± 20	0.6	0.003
CO (mL/min)	5.1 ± 1.4	5.1 ± 1.7	0.7	0.001
CI (mL/min/m^2^)	2.8 ± 0.8	2.7 ± 0.8	0.4	0.27 (*)
TAPSE (mm)	23.0 ± 3.0	23.0 ± 3.7	0.9	0.1
PASP (mmHg)	22.0 ± 3.0	25.0 ± 7.0	0.0001	0.0001
mPAP (mmHg)	15.4 ± 3.1	17.2 ± 4.2	0.0001	0.0001
PASP/CI	4.7 ± 2.2	10.2 ± 4.3	0.0001	0.0001 (*)
TAPSE/PASP (mm/mmHg)	1.2 ± 0.4	1.06 ± 0.3	0.04	0.0001
RVOT-AccTime (ms)	143 ± 27	127 ± 26	0.0001	0.0001
PVR (WU)	1.27 ± 0.4	1.6 ± 0.6	0.0001	0.008

Abbreviations: CI, left ventricular cardiac output index; CO, left ventricular cardiac output; AccTime, acceleration time; E/A, transmitral flow ratio; E/E’, E wave and E’ wave on TDI ratio; IVSD, interventricular septum in systole; LAVi, left atrial volume index; LV left ventricular mass; LVEF, left ventricular ejection fraction; LVIDD, left ventricular internal diameter in diastole; LVM, left ventricular mass; LVMI, left ventricular mass index; mPAP, mean pulmonary artery pressure; PASP, pulmonary arterial systolic pressure; PVR, pulmonary vascular resistances; PWTD, posterior wall thickness in diastole; RVOT-AccTime, acceleration time on RVOT (right ventricular outflow tract); RWT, relative wall thickness; SV, left ventricular stroke volume; TAPSE, tricuspid annular systolic excursion; TAPSE/PASP, tricuspid annular systolic excursion/ pulmonary systolic arterial. (*) no BSA.

**Table 3:** Difference between normotensive and hypertensive subjects at peak stress test.

	Normotensive *n* = 145	Hypertensive *n* = 155	*t*-Test	ANOVA Adjusted by Age, Sex, BSA
Watts (peak)	133 ± 38	104 ± 35	0.0001	0.0001
Time Exe (min)	10.4 ± 3.0	8.4 ± 2.8	0.0001	0.0001
SBP (mmHg)	188 ± 29	165 ± 24	0.0001	0.0001
DBP (mmHg)	96 ± 17	91 ± 13	0.047	0.02
HR (bpm)	138 ± 18	123 ± 19	0.0001	0.0001
LVEF (%)	71 ± 8.1	69 ± 11	0.09	0.0001
E/E’	6.6 ± 2.9	10.7 ± 5.5	0.0001	0.0001
SV (mL)	89 ± 20	86 ± 26	0.1	0.007
CO peak-rest (mL/min)	7.2 ± 2.8	5.2 ± 3.5	0.0001	0.0001
CO (mL/min)	12.5 ± 3.1	10.6 ± 3.6	0.0001	0.0001
CI (mL/min/m2)	6.6 ± 1.7	5.6 ± 1.8	0.0001	0.0001 (*)
TAPSE (mm)	28.3 ± 3.4	28.4 ± 5.2	0.7	0.0001
PASP (mmHg)	35.4 ± 9.4	47.3 ± 13.9	0.0001	0.0001
PASP peak-rest	13.6 ± 9.1	22.7 ± 13.7	0.0001	0.0001
mPAP (mmHg)	23 ± 5.8	31 ± 8.5	0.0001	0.0001
PASP/W	3.1 ± 1.4	5.1 ± 2.4	0.0001	0.0001
PASP/CI	5.8 ± 3.4	9.4 ± 4.6	0.0001	0.0001 (*)
TAPSE/PASP (mm/mmHg)	0.94 ± 0.2	0.65 ± 0.2	0.0001	0.0001
RVOT-AccTime (ms)	142.8 ± 33	116 ± 24	0.0001	0.0001
PVR (WU)	1.38 ± 0.5	1.74 ± 0.5	0.0001	0.0001

(*) no BSA.

**Table 4:** Hypertensive patient characteristics divided by age.

	Group 1 <51 Years *n* = 28	Group 2 >51 and <66 Years *n* = 65	Group 3 >66 Years *n* = 62	ANOVA	ANOVA Adjusted by Age, Sex, BSA
LVIDD (mm)	47.0 ± 3.9	48.0 ± 7.0		0.1	0.0001
Age (years)	43 ± 8	59.6 ± 4.4	73.5 ± 4.4	0.0001	
M/F	24/4	48/17	41/21	0.1	-
BMI	27 ± 5	28.4 ± 4	27 ± 3.8	0.2	-
BSA (m^2^)	1.95 ± 0.2	1.9 ± 0.2	1.83 ± 0.2	0.004	-
SBP (mmHg)	142 ± 22	136 ± 21	136 ± 18	0.3	0.0001
DBP (mmHg)	87.9 ± 13	83 ± 12	78.4 ± 8	0.002	0.0001
HR (bpm)	69.9 ± 13	74 ± 12	73 ± 13	0.4	0.002
Therapy (no/yes)	15/13	15/50	4/58	0.0001	0.0001 (*)
Hemoglobin (dL/L)	15 ± 1.7	14.4 ± 1.4	13.8 ± 1.9	0.02	0.0001
Creatinine (mg/dL)	0.98 ± 0.17	1.01 ± 0.19	1 ± 0.3	0.8	0.5
Glucose (mg/dL)	102 ± 22	124 ± 46	120 ± 41	0.1	0.11
Tot Chol (mg/dL)	190 ± 45	173 ± 62	157 ± 47	0.08	0.09
HDL (mg/dL)	51 ± 11	49 ± 11	50 ± 13	0.5	0.6
Triglycerides (mg/dL)	104 ± 59	137 ± 68	102 ± 49	0.6	0.018

Abbreviations: M/F, male/female; BMI, body mass index; BSA, body surface area; DBP, diastolic blood pressure; HR, heart rate; SBP, systolic blood pressure. No adjusted by therapy (*).

**Table 5:** Baseline echocardiographic characteristics in hypertensive patients divided into 3 groups of age.

	Group 1 ≤51 Years *n* = 28	Group 2 >51 and <66 *n* = 65	Group 3 ≥66 Years *n* = 61	ANOVA *p*	ANOVA Adj by Sex BSA, Therapy
LVIDD (mm)	49 ± 4.7	48 ± 8.5	47 ± 6.4	0.3	0.1
IVSD (mm)	9.6 ±2.0	10.4 ± 2.3	10.8 ± 1.7	0.036	0.025
PWTD (mm)	9.4 ± 2	9.1 ± 1.7	9.3 ± 1.5	0.1	0.9
LVM (gr)	176 ± 44	177 ± 89	171 ± 55	0.2	0.09
LVMI (gr/m^2^)	89 ± 20	91 ± 40	93 ± 25	0.1	0.1 (*)
RWT	0.39 ± 0.1	0.39 ± 0.1	0.40 ± 0.09	0.6	0.3
LVEF (%)	62 ± 8.5	64 ± 8	61 ± 9	0.07	0.6
LAi (vol/BSA)	21 ± 5.6	23.6 ± 9.9	29.4 ± 13	0.006	0.9 (*)
E/A	1.5 ± 0.7	1.2 ± 0.3	1.03 ± 0.3	0.0001	0.1
E/E’	6.7 ± 2.2	8.5 ± 3.2	10 ± 2.9	0.0001	0.0001
SV (mL)	69 ± 18	73 ± 23	69 ± 18	0.001	0.001
CO (L/min)	4.8 ± 1.5	5.4 ± 1.7	5.0 ± 1.7	0.8	0.03
CI (mL/min/m^2^)	2.5 ± 0.8	2.7 ± 0.8	2.7 ± 0.9	0.5	0.2 (*)
TAPSE (mm)	23.5 ± 3.7	23 ± 3.2	23 ± 4.2	0.7	0.001
PASP (mmHg)	22.2 ± 5.9	24.2 ± 5.7	26.6 ± 8.1	0.017	0.7
mPAP (mmHg)	15.9 ± 3.4	17.8 ± 3.7	17.6 ± 5	0.036	0.28
PASP/CI	9.7 ± 3.2	9.7 ± 3.9	10.9 ± 5.0	0.4	0.7 (*)
TAPSE/PASP (mm/mmHg)	1.1 ± 0.3	1.07 ± 0.3	1.02 ± 0.3	0.3	0.07
RVOT-AccTime (ms)	132 ± 23	127 ± 24	124 ± 26	0.09	0.2
PVR (WU)	1.61 ± 0.6	1.6 ± 0.5	1.6 ± 0.6	0.8	0.4

Abbreviations: AccTime, acceleration time; CI, left ventricular cardiac output index; CO, left ventricular cardiac output; E/A, transmitral flow ratio; E/E’, E wave and E’ wave on TDI ratio; IVSD, interventricular septum in systole; LAVi, left atrial volume index; LVEF, left ventricular ejection fraction; LVIDD, left ventricular internal diameter in diastole; LV left ventricular mass; LVMI, left ventricular mass index; mPAP, mean pulmonary artery pressure; PASP, pulmonary arterial systolic pressure; PWTD, posterior wall thickness in diastole; RWT, relative wall thickness; PVR, pulmonary vascular resistances; RVOT-AccTime, acceleration time on RVOT (right ventricular outflow tract); SV, left ventricular stroke volume; TAPSE, tricuspid annular systolic excursion; TAPSE/PASP, tricuspid annular systolic excursion/pulmonary systolic arterial. (*) no BSA.

**Table 6:** Hypertensive patient characteristics at peak stress divided by age.

	Group 1 ≤51 Years *n* = 28	Group 2 >51 and <66 *n* = 65	Group 3 ≥66 Years *n* = 62	ANOVA	ANOVA Adj by Sex, BSA, Therapy
Watts (peak)	120 ± 33	100 ± 32	93 ± 35	0.0001	0.001
Time Exe (min)	5.2 ± 1.2	4.3 ± 1.3	3.6 ± 1.3	0.0001	0.001
SBP (mmHg)	175 ± 28	169 ± 27	158 ± 19	0.1	0.2
DBP (mmHg)	104 ± 15	95 ± 14	84 ± 8	0.0001	0.5
HR (bpm)	133 ± 16	127.3 ± 16	113 ± 19	0.0001	0.001
LVEF (%)	74 ± 9.5	69 ± 10	65 ± 12	0.01	0.001
E/E’	7.5 ± 2.0	9.7 ± 6.0	13.0 ± 4.6	0.005	0.0001
SV (mL)	89.0 ± 29.0	88.7 ± 25.0	82.0 ± 27.0	0.4	0.0001
CO peak-rest	7.1 ± 3.8	5.5 ± 3.0	4.4 ± 3.3	0.005	0.0001
CO (L/min)	12.0 ± 3.5	11.3 ± 3.4	9.3 ± 3.5	0.0001	0.0001
CI (L/min/m2)	6.1 ± 1.7	5.8 ± 1.7	5.5 ± 1.7	0.007	0.0001 (*)
TAPSE (mm)	32 ± 5.3	28 ± 4.8	27 ± 4.8	0.0001	0.0001
PASP (mmHg)	51 ± 16	43 ± 13	49 ± 12	0.01	0.0001
PASP/CI	8.1 ± 2.9	7.9 ± 3.9	11.1 ± 5.0	0.001	0.016
mPAP (mmHg)	34 ± 10.0	28 ± 8.0	32 ± 7.1	0.006	0.0001
TAPSE/PASP (mm/mmHg)	0.66 ± 0.2	0.71 ± 0.2	0.56 ± 0.2	0.0001	0.059
AccTime (ms)	127 ± 24	118 ± 20	110 ± 2	0.04	0.003
PVR (WU)	1.73 ± 0.5	1.70 ± 0.5	1.79 ± 0.5	0.8	0.2

Abbreviations: AccTime, acceleration time; CI, left ventricular cardiac output index; CO, left ventricular cardiac output; E/E’, E wave and E’ wave on TDI ratio; LVEF, left ventricular ejection fraction; LV left ventricular mass; mPAP, mean pulmonary artery pressure; PASP, pulmonary arterial systolic pressure; PVR, pulmonary vascular resistances; RVOT-AccTime, acceleration time on RVOT (right ventricular outflow tract); SV, left ventricular stroke volume; TAPSE, tricuspid annular systolic excursion; TAPSE/PASP, tricuspid annular systolic excursion/pulmonary systolic arterial. (*) no BSA.

**Table 7:** Evaluation of hemodynamic parameters at peak exercise tested by mixed model.

	Fixed Factors	Adjustment	*p* Value
E/E’ PEAK exercise	Hypertensive status	Gender	0.2
	Age	Therapy	0.7
	Hypertensive status * Age	E/E’ at baseline	0.0001
TAPSE PEAK	Hypertensive status	Gender	0.0001
	Age	Therapy	0.8
	Hypertensive status * Age	TAPSE at baseline	0.007
PASP PEAK	Hypertensive status	Gender	0.0001
	Age	Therapy	0.002
	Hypertensive status * Age	PASP at baseline	0.15
TAPSE/PASP PEAK	Hypertensive status	Gender	0.0001
	Age	Therapy	0.014
	Hypertensive status * Age	TAPSE/PASP baseline	0.66
CO PEAK	Hypertensive status	Gender	0.6
	Age	Therapy	0.012
	Hypertensive status * Age	CO at baseline	0.53

Abbreviations: CO, left ventricular cardiac output; PASP, pulmonary arterial systolic pressure; TAPSE, tricuspid annular systolic excursion; TAPSE/PASP, tricuspid annular systolic excursion/ pulmonary arterial systolic pressure. * = interaction (hypertensive state and age).

## Data Availability

The data that support the findings of this review are openly available in the References section.

## Supplementary Materials

The following supporting information can be downloaded at: https://www.mdpi.com/article/10.3390/jcm11020451/s1, Table S1: Trend of hemodynamic parameters at baseline and at peak exercise in normotensive and hypertensive subjects.
