# Supplementary material for: Right Heart Pulmonary Circulation Unit Response to Exercise in Patients with Controlled Systemic Arterial Hypertension: Insights from the RIGHT Heart International NETwork (RIGHT-NET)
Source: J Clin Med. 2022 Jan 17;11(2):451. doi: 10.3390/jcm11020451 (PMC8778233; doi:10.3390/jcm11020451)
Supplement: Supplementary file 1 [file jcm-11-00451-s001.zip › jcm-1496861-SI.pdf]

Supplementary Table S1. Trend of hemodynamic parameters at baseline and at peak exercise in normotensive and hypertensive subjects

	**Normotensives**	**Hypertensives**	**p**
E/E′	**Baseline**	**Baseline**	(T-Test)
All group	7.3±2.5	8.8±3.2	0.0001
Group1	6.3±1.7	6.7±2.2	0.3
Group2	7.2±2.3	8.5±3.2	0.026
Group3	9.2±3.9	10.2±2.9	0.16
E/E′	**Peak exercise**	**Peak exercise**	
All group	6.6±2.9	10.7±5.5	0.0001
Group1	7.1±2.3	7.5±2.1	0.4
Group2	6.2±3.4	9.7±6.4	0.004
Group3	6.2±3	13±4.6	0.0001
PASP (mmHg)	**Baseline**	**Baseline**	
All group	22±3.0	25±7	0.0001
Group1	20.7±5.1	22.2±4.5	0.2
Group2	22.8±4.5	24.2±5.7	0.17
Group3	23.9±5.8	26.5±8.1	0.08
PASP (mmHg)	**Peak**	**Peak**	
All group	35.4±9.4	47.3±13.9	0.0001
Group1	32.5±7	52.5±16.7	0.0001
Group2	35.0±8.1	43.3±13.6	0.0001
Group3	40.7±11.9	49.2±11.8	0.001
CO (L/min)	**Baseline**	**Baseline**	
All group	5.1±1.4	5.1±1.7	0.7
Group1	5.3±1.4	4.8±1.6	0.1
Group2	4.9±1.5	5.4±1.7	0.1
Group3	4.9±1.3	5±1.7	0.8
CO (L/min)	**Peak**	**Peak**	
All group	12.2±3.1	10.7±3.6	0.0001
Group1	13±2.7	12.3±3.6	0.3
Group2	12.3±3	11.3±3.4	0.1
Group3	11.0±3.4	9.3±3.5	0.02
TAPSE	**Baseline**	**Baseline**	
All group	22.9±3.1	23±3.7	0.9
Group1	23.2±2.8	23.5±3.7	0.7
Group2	22.8±3.3	23.02±3.2	0.7
Group3	22.5±3.4	22.7±4.2	0.8
TAPSE	**Peak**	**Peak**	
All group	28.4±3.5	28.4±5.2	0.8
Group1	27.8±3.3	32±5.4	0.0001
Group2	28.9±2.9	28.3±4.8	0.4

Group3	28.8±4.4	26.8±4.7	0.053

TAPSE/PASP	**Baseline**	**Baseline**	
All group	1.16±0.4	1.06±0.3	0.04
Group1	1.25±0.5	1.14±0.3	0.3
Group2	1.07±0.3	1.07±0.3	0.9
Group3	1.1±0.4	1.02±0.39	0.8

TAPSE/PASP	**Peak**	**Peak**	
All group	0.94±0.29	0.65±0.21	0.0001
Group1	1.0±0.25	0.66±0.18	0.0001
Group2	0.95±0.21	0.71±0.22	0.0001
Group3	0.83±0.38	0.55±0.18	0.0001

Abbreviations: CO, left ventricular cardiac output; HT, hypertension; PASP, pulmonary arterial systolic pressure; TAPSE, tricuspid annular systolic excursion; TAPSE/PASP, tricuspid annular systolic excursion/pulmonary arterial systolic pressure.
